# Nurses’ experiences of the role of organizational and environmental factors in the development of love of the profession: a qualitative study

**DOI:** 10.1186/s12912-022-01117-4

**Published:** 2022-11-29

**Authors:** Shahnaz Bolandian-Bafghi, Mohsen Adib-Hajbaghery, Mitra Zandi

**Affiliations:** 1 Research Center for Nursing and Midwifery Care, Shahid Sadoughi University of Medical Sciences, Yazd, Iran; 2 Trauma Nursing Research Center, Kashan University of Medical Sciences, Kashan, Iran; 3 Medical Surgical Nursing Department, School of Nursing and Midwifery, Shahid Beheshti University of Medical Sciences, Tehran, Iran

**Keywords:** Content analysis, Love, Love of the profession, Nurse, Nursing profession, Qualitative study

## Abstract

**Background:**

Love of the profession has significant relationship with nurses’ job motivation and care quality. However, there is limited information about organizational and environmental factors affecting love of the profession among nurses.

**Aim:**

This study aimed at exploring nurses’ experiences of the role of organizational and environmental factors in the development of love of the profession.

**Methods:**

This qualitative study was conducted in 2020–2021 using the conventional content analysis approach. Participants were Fifteen Iranian nurses with deep love of the profession, nursing instructors, and nursing managers purposefully selected from different healthcare and academic settings in seven large cities of Iran. Data were gathered via semi-structured interviews and were analyzed via the conventional content analysis method proposed by Graneheim and Lundman.

**Results:**

Organizational and environmental factors affecting the development of love of the profession were grouped into three main categories: The social context of the profession (with two subcategories), family support (with two subcategories), and organizational health (with four subcategories). Subcategories were respectively historical context of the profession, the evolving context, emotional family support, instrumental family support, quality of interpersonal relationships in the organization, level of organizational justice, level of authority delegation to nurses, and level of organizational support.

**Conclusion:**

Different organizational and environmental factors can affect nurses’ love of the profession development. Improving public image of nursing, providing nurses with stronger support, improving organizational climate, and strengthening interpersonal relationships in healthcare settings are recommended to develop nurses’ Love of the profession.

## Introduction

Nursing is a profession because nurses have autonomy, professional commitment, specialty, knowledge and skills, value system, and academic education [1]. The presence of values is one of the key characteristics of nursing that makes it a profession [2]. Florence Nightingale, the founder of modern nursing, states that nursing is not a scientific knowledge and a technical skill; rather, it is a profession that is based on human values [3, 4] such as love for human beings and for providing care to them [5]. She also highlights that this love for human beings is the base of love of the profession (*lop*) among nurses [6]. By reconsidering the power of love in nursing, nurses are reminded of what truly is valuable and thus bring back the light and love within themselves and serve people with affection and strength [7].

There are many different definitions for *lop*. Some scholars described *lop* as feelings of interest, concern, respect, understanding, responsibility, benevolence, altruism, and dedication [6, 8]. Some scholars also used *lop*-related concepts, such as work engagement, to define *lop*, while work engagement is a positive subjective approach to the profession characterized by attributes such as power, dedication, and absorption [9, 10]. According to what was said, in this study, *lop* is defined as an abstract concept that emerges and manifests itself with the appearance of symptoms such as self-sacrifice, benevolence, altruism, and sense of responsibility in the nurse’s practice.

*lop* has different effects on both nurses and their clients. It deepens nurses’ understanding of patients and their sufferings [11], guides nurses’ behaviors and actions, improves the quality of their care services, facilitates patient recovery, and enhances patient satisfaction [6]. Nurses with *lop* use their whole physical, cognitive, and emotional abilities at work, while limited *lop* is associated with the separation of physical, cognitive, and emotional abilities from professional activities and the separation of nurses from their professional roles [12].

Although nursing textbooks emphasize care based on the nursing process, in clinical education, Iranian students are generally trained in a task-oriented manner, and are mainly prepared to carry out physicians’ orders [13]. Therefore, nursing is physician-based, and a pivot routine ( they mostly perform some technical and routine care) is one of the most important characteristics of Iranian nurses [14]. The reasons mentioned above generally demotivate most nurses and make them less interested in their profession.

Earlier, we examined the factors contributing to the development of the *lop* and have reported that the public perception of the profession, educational variables, characteristics of the profession, and nurses’ self-evaluation effectively develop the *lop* in nurses. Although in that report several nurses cited the impact of social factors on the development of the *lop* [15]. However, the environmental and organizational factors affecting the development of *lop* in nurses have not been assessed. The findings of this study raise the question for researchers that apart from the profession’s public image, whether or not other environmental and social factors affect the *lop* in nurses. The previous study’s findings only emphasize the effect of society’s public image on love of the profession and have no answers to the questions above. Therefore, the present study tries to elucidate these factors. In this study, the researchers tried to limit their questions to the influences of environmental and organizational factors and examin their impact on the profession’s love.

To the best of our knowledge, none of the previous studies explored factors affecting *lop*, particularly organizational and environmental factors. Most studies in this area explored factors affecting *lop*-related concepts such as work engagement. For example, a study showed that 37% of students developed interest in nursing due to environmental factors [16]. Another study reported that work-family enrichment improved nurses’ mood, reduced their perceived stress, increased their resistance to stressors, and thereby, enhanced their job satisfaction and lowered their turnover rate [17]. A study also showed that transformational leadership and structural empowerment were the significant predictors of nurses’ work engagement [18]. Similarly, a study reported that nursing managers had significant roles in improving nurses’ work engagement, while payments and benefits were not the principal factors in nurses’ work engagement [19].

Given the paucity of information about factors affecting *lop*, the present study was conducted to narrow this gap.

### Aim of the study

The aim of the study was to explore nurses’ experiences of the role of organizational and environmental factors in the development of *lop*.

## Methods

This qualitative content analysis study was conducted in 2020–2021 using the conventional content analysis approach. In qualitative content analysis, manifest and latent concepts are extracted from participants’ descriptions and then are coded, abstracted, and categorized to develop main categories or themes [20].

### Participants

Participants were Iranian nurses with deep *lop*, nursing instructors, and nursing managers. They were purposefully selected from different healthcare settings and universities in seven cities of Iran, namely Yazd, Isfahan, Kashan, Tehran, Mashhad, Birjand, and Kerman. Sampling was performed with maximum variation in order to include a wide range of experiences and viewpoints in the study. The inclusion criteria were a desire to attend the study, clinical work experienceof at least two years, popularity as a nurse with deep *lop* among colleagues, the ability to share *lop*-related experiences, and being able to share their experiences in detail. Three nurses refused participation due to factors such as the risk of COVID-19 transmission, heavy workload, concerns over childrearing and their children’s education, and fatigue. Having explained the objectives of the research to the nurse managers, they were asked to introduce those nurses who are enthusiastic and love their profession. They introduced such nurses to the researcher with respect to characteristics they believed are the key to choose: a sense of responsibility, high performance, proper behavior, resilience, passion and a sense of initiation. The participants were also asked to introduce someone they knew who loved the profession. Before the researcher started the main study, the interview guide was pilot-tested on two nurses to ensure the consistency of the questions’ meanings, and that the questions were clear and comprehensible. These two nurses did not participate in the main study, and their interviews were discarded.

### Data collection

In-depth semi-structured interviews were conducted to collect these data. Initially, some broad questions were used to establish appropriate communication with participants and gain their trust. Then, the following questions were used for data collection, “Have environmental factors affected your *lop*?” and “Can you explain about your experiences of the effects of environmental factors on your *lop*?” Moreover, questions such as “May you explain more?” and “What led to this?” were used to encourage participants to provide more details about their experiences. The place and the time of interviews were arranged according to participants’ preferences and all interviews were conducted in their lounge. Interviews were held by the second author and lasted 30–90 min. The interview process were continued up to data saturation. Saturation was ensured when no new conceptual codes were obtained from interviews as well as aspects of organizational and environmental factors were explored [21]. A second interview was conducted with some of the participants if the researcher encountered any ambiguities or questions while reading and analyzing the interviews. In this way, participants number 1, 2, 3 and 13 were interviewed twice. Saturation was achieved after Nineteen interviews with Fifteen participants. All interviews were audio-recorded using an MP3 player.

### Data analysis

Data were analyzed via the conventional content analysis method proposed by Graneheim and Lundman [20]. Each interview was listened to for several times and was transcribed word by word using the Microsoft Word. Then, each interview was considered as the unit of analysis and was perused several times to immerse in the data. Thereafter, important words and sentences which were relevant to the study aim were identified and coded and the codes were categorized according to their similarities. Developed categories were revised, further developed, and combined with each other during the analysis of each new interview and thereby, main subcategories and categories were identified. Data analysis was managed via the MAXQDA 10 software.

### Trustworthiness

Trustworthiness was established using the criteria proposed by Guba and Lincoln, namely credibility, dependability, confirmability, and transferability [22]. Credibility was established through member checking, peer checking, prolonged engagement with these data, and immersion in the data. Dependability was also established through peer checking. In peer checking, three nursing scholars external to the study with considerable experience in qualitative research assessed and confirmed the accuracy of data analysis. For confirmability, A part of the full text of the interview with the initial codes was reviwed by the participants and the degree of congruence of the researcher’s idea of the data with the opinion of the participants was compared. The suggested points were taken into consideration, and in case of ambiguity, the participant was asked for further explanation. Transferability was ensured through providing thick descriptions of the processes of data collection and analysis. Moreover, two nurses with *lop* who were external to the study were asked to assess the similarity between our findings and their own experiences. They confirmed that our findings were similar to their experiences. In addition, COREQ checklist was used to structure the reporting of the study.

## Findings

Participants were nine female and Six male nurses with *lop*, nursing instructors, and nursing managers. Their age and work experience ranged 30–59 and 7–30 years. Four participants had master’s degree and the remaining participants had bachelor’s degree. Six participants were hospital nurses, two were head nurses, two were supervisors, two were nursing instructors, one was infection control staff, one was relief staff and one was manager. All of them had clinical work experience and four of them had the experience of teaching to nursing students. Table 1 shows participants’ characteristics.

**Table 1:** Participants’ demographic characteristics

Demographic characteristics		Frequency	Percent
Gender	Male	6	40%
	Female	9	60%
Age(year)	< 35 years	2	13%
	35–45 years	7	47%
	> 45 years	6	40%
Degree	Bachelor’s	11	73%
	Master’s	4	27%
Work experience (Years)	< 10 years	2	13%
	10–15 years	3	20%
	> 15 years	10	67%
	Total	15	100%

In total, 109 conceptual codes were generated during data analysis which were categorized into eight subcategories and three main categories. The main categories were The social context of the profession, family support, and organizational health (Table 2).

**Table 2:** The main categories and subcategories of organizational and environmental factors in love of profession development

Main Categories	Subcategories
The social context of the profession	Historical context of the profession
	The evolving context of the profession
Family support	Emotional family support
	Instrumental family support
Organizational health	Quality of interpersonal relationships in the organization
	level of organizational justice
	Level of authority delegation to nurses
	Level of organizational support

### The social context of the profession

The social context of the profession was one of the main environmental factors affecting *lop* development. It reflects how the nursing appears to people and what mental ideas or judjments the public have about it. The two subcategories of this category were historical context of the profession and the evolving context.

#### Historical context of the profession

The historical context of the profession refers to the common view of society in the nursing profession. This context and the quality of public respect for nursing can considerably affect nurses’ *lop*. Our participants noted that they felt deeper *lop* whenever they were respected and valued for their profession. Contrarily, stereotypical or negative images of nursing negatively affected their *lop*. They reported culture, religion, and tradition in the community as a compelling social context for creating and developing a profession’s love.

*As a man, I entered the nursing profession in a bad period because the culture of Iranian society hardly accepted men as nurses. ( p.6).*

*People respected me whenever they found I am a nurse. This increased my interest in nursing (P. 10).*

*I had many good suitors. However, all of them changed their minds when I entered nursing. Why? Because they had a poor image of nursing. They believed that nurses are unrestrained, corrupt and shameless. Such things cause me not to love nursing a lot (P. 14).*

#### The evolving context

Participants noted that significant events such as war, earthquake, and the coronavirus 2019 (COVID-19) pandemic have suddenly changed the public image and value of nursing. They noted that nurses’ dedication to saving veterans’ lives during the war, earthquake victims, and patients’ lives during the COVID-19 pandemic had significant positive effects on society’s view of the profession and has caused a sudden change in society towards the nursing profession.

*Nurses’ attempt to save veterans’ lives changed public image of nursing and hence, people no longer have negative image or attitude towards nursing (P. 5).*

*Public mind-set towards us has considerably improved after the prevalence of the COVID-19. People’s respect for us has made me have better feelings towards nursing (P. 9).*

### Family support

The experiences of some participants showed that family had significant effects on their *lop*. Family support for them caused them not to feel loneliness in the midst of their responsibilities. Strong family support fostered in participants the belief that their profession as well as themselves were accepted by family members and gave them sense of worthiness. Moreover, family support increased their success in professional and familial affairs and thereby, improved their work engagement. This category had two subcategories, namely emotional family support and instrumental family support.

#### Emotional family support

Nursing, particularly clinical nursing, is a difficult profession due to different work shifts, long work hours, and heavy workload. Therefore, nurses need strong support to be able to effectively perform their professional roles. The experiences of some participants showed that they had such support. Emotional support by family increased participants’ professional and familial success and thereby, improved their job motivation, professional self-image, professional interest, and *lop*. The different aspects of emotional family support were feeling proud of a nurse family member, husband’s good attitude towards nursing, and family encouragement, acceptance, and understanding.

*My husband has a very good attitude towards nursing. He is just like me. He says that nursing is your job, your duty, the thing that you was interested in and studied for it. He considers me important for society (P. 1).*

However, the experiences of some participants showed that their families not only had no good attitude towards nursing, but also encouraged them to quit the profession.

*My family members don’t accept nursing at all and recommend me to quit it (P. 14).*

#### Instrumental family support

Multiple familial and professional responsibilities, particularly among female nurses, together with others’ expectations exert significant physical and mental effects on nurses. Our participants’ experiences showed that instrumental support by family reduced their role conflicts, fatigue, and strain, improved their energy for professional activities, increased their professional success, and thereby, fostered greater *lop* among them. The different types of instrumental support by family members were help in childrearing, help in children’s school affairs, and help in performing household activities.

*My husband has arranged his work hours according to my work hours so that he takes care of children when I’m at work. Therefore, I have no concern over children and do my job with greater concentration (P. 7).*

### Organizational health

Organizational health and suitability can also affect *lop* among nurses. Participants’ experiences showed that interpersonal relationships in the organization, organizational justice, personal power and autonomy in organization, and organizational support for staff had significant roles in developing their *lop*. The four subcategories of this category were the quality of interpersonal relationships in the organization, level of organizational justice, level of authority delegation to nurses, and level of organizational support.

#### Quality of interpersonal relationships in the organization

Some participants highlighted that a friendly organizational atmosphere and appropriate interpersonal communications at work provide nurses with better feelings and greater peace.

*The work atmosphere in our setting was very friendly when I started my work. For example, our head nurse had a very good conduct*. *He helped us a lot in our work and was kind at the same time*. *This increased my interest (P. 3).*

On the other hand, paternalistic approach of authorities can reduce positive emotions in the workplace and thereby, reduce nurses’ interest in work.

*Some authorities treat nurses, particularly novice nurses, as they are soldiers at a military base. Instead of a rigid conduct from the very beginning, we need to make novice nurses interested in work (P. 8).*

#### Level of organizational justice

Most participants reported organizational justice as an influential factor on *lop*. Fair payments, competence-based appointments, and no discrimination among nurses can bring nurses senses of peace, security, and justice, gain nurses’ trust in organization, promote their professional and organizational commitment, and foster their *lop*.

*Justice, non-discrimination, and competence-based career advancement in the organization increase our professional interest (P. 10).*

*Injustice is the most disturbing thing for nurses. For example, during this COVID-19 pandemic, a doctor visits a patient just for half an hour and receives the salary of twelve hours work of a nurse for such visit. Such injustice reduces nurses’ motivation (P. 6).*

#### Level of authority delegation to nurses

The level of authority delegation to nurses was another organizational factor affecting *lop*. By authority delegation, participants meant autonomy at work, decision making power, latitude, and the right to freely share ideas. Participants noted that based on their professional knowledge and skills, nurses need autonomy and latitude at work and highlighted that autonomy and latitude give nurses sense of power and self-confidence and increase their motivation for professional practice.

*Greater autonomy and latitude for nurses reduce their dependence on physicians, give them sense of power, and help them work more independently, which in turn improve their interest and motivation (P. 9).*

Contrarily, authorities’ paternalistic behaviors and nurses’ limited perceived autonomy and power give nurses sense of oppression and reduce their job motivation and interest in profession and organization.

*The approach of the Ministry of Health is paternalistic, in which power is with physicians. For example, the salary of a doctor is 100 times the salary of a nurse. Violation of nurses’ rights in this system reduces their motivation (P. 6).*

#### Level of organizational support

According to the participants, organizational support can also affect nurses’ *lop*. They noted that as staff of an organization establish emotional relationship with their organization, the level of tangible and intangible support by their organization and managers can affect their *lop* and professional interest.

*I was on a long evening-night shift. We had a critically-ill patient and were involved with the patient up to 06:00. I was extremely tired. My colleagues suggested me to take a rest. I took a rest from 06:00 to 06:30. This is not a legal time for rest in our hospital. When I returned the ward at 06:30, my colleagues said that the matron of hospital had been in the ward and considered a reward for me for that busy shift. Such value attachment to my work was very important to me. My manager did not criticize me for being on rest in an illegal time. This motivates staff and increases their belongingness to work (P. 4).*

On the other hand, lack of organizational support can reduce *lop*.

*When they assigned me night shifts, I told them I couldn’t work at night and asked them to reduce the number of my night shifts. However, they refused. Nobody understood me (P. 14).*

## Discussion

This study explored nurses’ experiences of the role of organizational and environmental factors in *lop* development. Therefore, it can be claimed that this study is one of the few studies performed in the field of *lop* in nurses. Participants’ experiences showed that the main organizational and environmental factors in *lop* development among nurses were the social context of the profession, family support, and organizational health.

The social context of the profession was one of the main environmental factors in developing *lop*. The social context of the profession indicates the ideas people have about nursing and affect the level of public respect for nursing and public understanding about it. In line with this finding, former studies reported that the social status of nursing significantly affects its development, social respect for it [23], nurses’ job motivation their job satisfaction, and their *lop *[24, 25]. However, our participants were dissatisfied with stereotypical images of nursing and considered them as factors with negative effects on their job motivation and professional interest. Even after many years of men’s entrance in nursing, most people in Iran consider nursing as an inappropriate profession for men. This attitude negatively affects men’s motivation to enter nursing [26]. Similarly, a study in East Africa showed that despite improvements in the public image of nursing, some people still believed that nurses were rude, cruel, thieve, and handmaiden [27]. Nonetheless, we found that significant events such as war, earthquake, and the COVID-19 pandemic can affect public attitude and social context towards nursing. Two previous studies also reported significant improvements in public attitude towards nursing after wars [28, 29]. During the COVID-19 pandemic, people around the world also admired nurses, appreciated their practice, and showed love for them [30], which resulted in positive feelings such as proud and honor among nurses [31].

The second main factor in *lop* development among nurses was family support. Previous studies also reported that nurses’ familial problems and limited family support can negatively affect their professional practice, job satisfaction, and job motivation [32, 33], and highlighted the significant positive effects of emotional and instrumental support by family members on nurses’ performance at home and work [34]. These findings denote that family support facilitate *lop* development, while limited family support can result in family-work conflicts, low job motivation, and intention to quit nursing.

Organizational health was the third main factor in *lop* development. Our findings showed that interpersonal relationships at work, organizational justice, organizational support, and the level of authority delegation to nurses were the main organizational factors affecting nurses’ *lop*. A supportive work environment can reduce nurses’ role ambiguities, empower them to use their skills and establish effective interpersonal relationships with their colleagues, make them value non-material aspects of work more than its material aspects, and improve their professional commitment and job satisfaction [35]. Nurses’ effective interactions with each other and with their managers significantly improve their organizational attachment [36, 37]. Moreover, organizational support, friendly atmosphere at work, and close relationships with colleagues have significant positive relationships with nurses’ work engagement [38]. A study also reported that leader-nurse interaction had significant relationship with nurses’ organizational commitment, professional practice [39], and work engagement [19]. Another study showed that organizational support had significant effects on healthcare workers’ organizational attachment and their intention to leave their professions [4]. Similarly, a study found that workplace empowerment had significant positive effects on nurses’ practice, behaviors, organizational commitment, and work engagement [40]. Furthermore, a study showed lower ambiguities, higher staff morale and productivity, greater organizational commitment and professional interest, and lower staff turnover in healthy organizations [41]. All these findings confirm the significant effects of organizational characteristics on *lop*. Nonetheless, there is no consensus over the effects of organizational factors on nurses. For example, a study found that salaries and benefits were not among the main predictors of work engagement [19]. Another study showed that nurse-peer interaction had no significant relationship with nurses’ professional practice [39]. These contradictions are attributable to the differences among studies respecting their settings, designs, and participants.

### Implications for nursing and health policy

Identifying the organizational-environmental factors behind the development of love for the nursing profession would help health care policymakers manipulate these factors and develop love and commitment toward their profession in nurses.The development of love for the profession in nurses will increase their professional commitment and the quality of nursing services, reduce their turnover, and ultimately, will improve the position of the nursing profession in society, as well as the level of public health.

## Conclusion

This study concludes that *lop* development among nurses is affected by different organizational and environmental factors, The social context of the profession, family support, and organizational health. Therefore, strategies such as correcting stereotypical public images of nursing, improving public status of nursing, providing nurses with stronger familial, organizational, and social support, improving organizational climate, and strengthening interpersonal relationships in healthcare organizations are recommended to improve nurses’ job satisfaction and motivation and develop their *lop*. *lop* development can in turn improve the quality of nursing care and positively affect public health.

### Recommendations

Future studies are recommended to develop and assess the effects of *lop* development strategies. Moreover, given the positive effects of the COVID-19 pandemic on public attitude towards nursing, qualitative studies are needed to further explore the effects of this pandemic on nurses’ *lop*.

### Limitations

Inaccessibility to a similar study (for comparison) was the limitations of this study.

## Data Availability

All the raw data (participants’ voice files and the texts of the interviews) will be confidential and cannot be provided to anyone. However, the codes emerged during the current study are available from the corresponding author on reasonable request.

## Abbreviation

*lop*: Love of the profession
